# Supplementary material for: Prognostic Factors for the Efficiency of Radiation Therapy in Dogs with Oral Melanoma: A Pilot Study of Hypoxia in Intraosseous Lesions
Source: Vet Sci. 2022 Dec 22;10(1):4. doi: 10.3390/vetsci10010004 (PMC9861487; doi:10.3390/vetsci10010004)
Supplement: Supplementary file 1 [file vetsci-10-00004-s001.zip › Supplemental figure.pptx]

## Slide 1
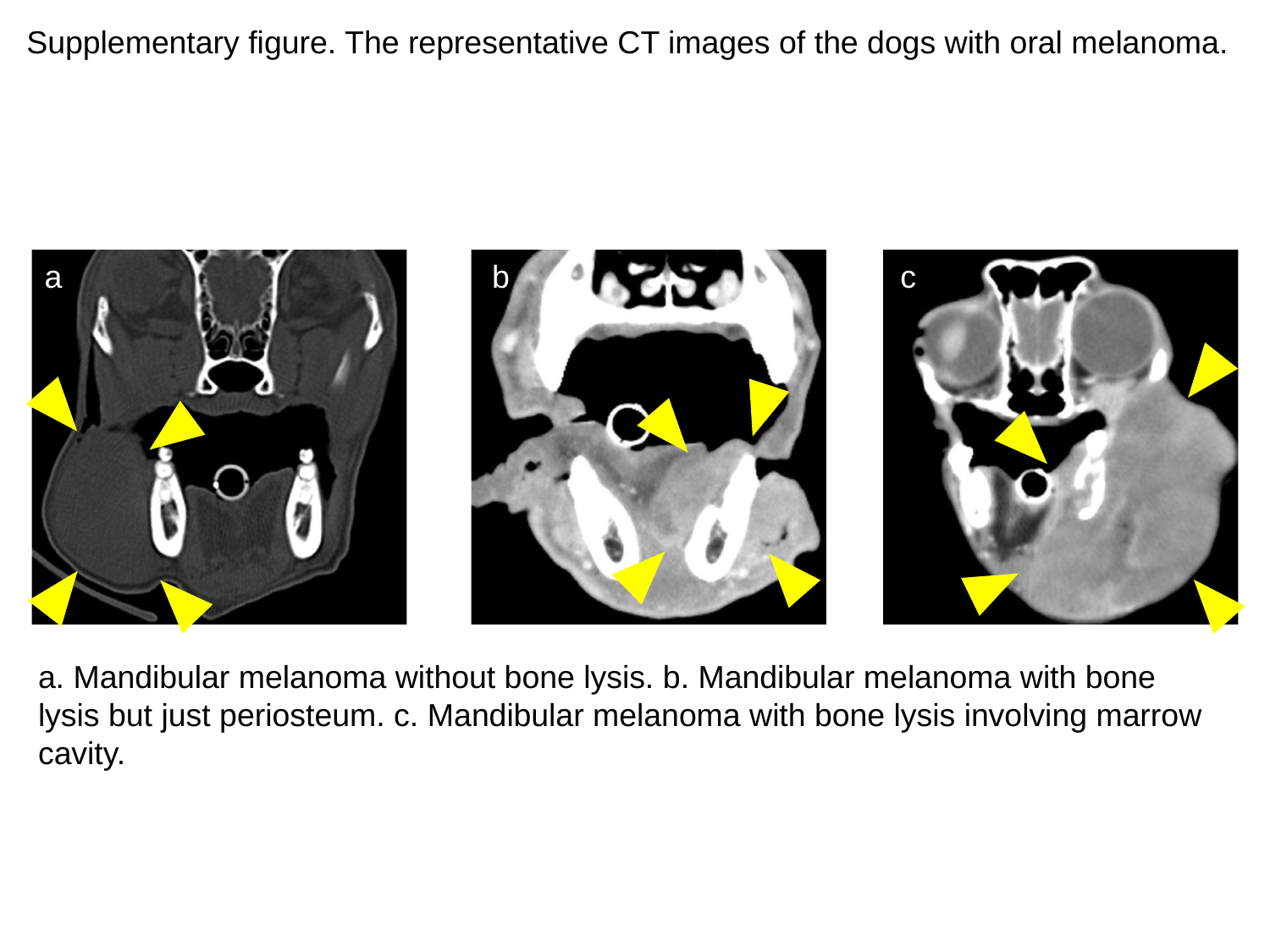

Supplementary figure. The representative CT images of the dogs with oral melanoma.
a
b
c
a. Mandibular melanoma without bone lysis. b. Mandibular melanoma with bone lysis but just periosteum. c. Mandibular melanoma with bone lysis involving marrow cavity.
